# Effect of Hydrogen Annealing on Performances of BN-Based RRAM

**DOI:** 10.3390/nano13101665

**Published:** 2023-05-18

**Authors:** Doowon Lee, Hee-Dong Kim

**Affiliations:** Department of Semiconductor Systems Engineering, and Convergence Engineering for Intelligent Drone, Institute of Semiconductor and System IC, Sejong University, 209, Neungdong-ro, Gwangjin-gu, Seoul 05006, Republic of Korea; dwlee@sejong.ac.kr

**Keywords:** boron nitride, hydrogen annealing, reliability, RRAM, aerospace

## Abstract

BN-based resistive random-access memory (RRAM) has emerged as a potential candidate for non-volatile memory (NVM) in aerospace applications, offering high thermal conductivity, excellent mechanical, and chemical stability, low power consumption, high density, and reliability. However, the presence of defects and trap states in BN-based RRAM can limit its performance and reliability in aerospace applications. As a result, higher set voltages of 1.4 and 1.23 V were obtained for non-annealed and nitrogen-annealed BN-based RRAM, respectively, but lower set voltages of 1.06 V were obtained for hydrogen-annealed BN-based RRAM. In addition, the hydrogen-annealed BN-based RRAM showed an on/off ratio of 100, which is 10 times higher than the non-annealed BN-based RRAM. We observed that the LRS changed to the HRS state before 10,000 s for both the non-annealed and nitrogen-annealed BN-based RRAMs. In contrast, the hydrogen-annealed BN-based RRAM showed excellent retention characteristics, with data retained up to 10,000 s.

## 1. Introduction

Non-volatile memory (NVM) devices have been used in aerospace and defense systems due to their ability to store data reliably in harsh environments, such as extreme temperatures, radiation, and mechanical shock [1]. In aerospace applications, NVM is used in various systems, including mission-critical applications, avionics, and data recorders. One of the primary requirements for NVM in aerospace applications is high reliability and endurance. This is because the data stored in these systems must remain intact over long periods, even in the face of extreme environmental conditions. For example, data recorders in aircraft must be able to withstand the forces of a crash and still be able to provide valuable data to investigators. Another critical requirement for NVM in aerospace applications is low power consumption. This is because many aerospace systems, such as satellites, and drones for aerospace applications, operate on limited power and cannot afford to waste energy on memory systems.

Early aerospace systems used magnetic core memory or magnetic tape for data storage, but these technologies were bulky, heavy, and had limited endurance. With the advent of semiconductor technology, NVM solutions became more feasible, and their benefits became apparent. In the 1980s, flash memory began to gain popularity in aerospace applications due to its low power consumption, high density, and reliable performance in harsh environments. One of the earliest applications of flash memory in aerospace was in the Space Shuttle program. The Space Shuttle used a variety of electronic systems, including avionics, data recorders, and guidance systems, all of which required reliable data storage. Flash memory was used in the data recorders and in some of the avionics systems, where its low power consumption and high endurance were particularly beneficial.

As computing technology advances, NVM technologies, such as phase-change random-access memory (PCRAM) [2], magnetoresistive RAM (MRAM) [3], ferroelectric RAM (FeRAM) [4], and resistive RAM (RRAM) [5], are gaining increasing attention in the field of computer memory. These technologies offer several advantages over traditional NVM solutions, including faster read and write speeds, lower power consumption, and greater endurance. PCRAM uses a material that can change between an amorphous and a crystalline state to store data. When the material is in its amorphous state, it has a high resistance, while in its crystalline state, it has a low resistance. The data is stored by changing the material between these two states. PCRAM has high endurance, low power consumption, and fast write speeds, but its read speed is relatively slow. MRAM is a non-volatile type of RAM that stores data bits using magnetic states instead of electrical charges. MRAM uses a pair of ferromagnetic metal plates separated by a thin insulating material layer to store data. The orientation of the two magnetic fields determines whether a binary bit is a 1 or 0. MRAM is inherently nonvolatile, and the magnetic states can be switched extremely fast and with no wear out. By combining the high speed of static RAM (SRAM) and the high density of DRAM, MRAM promises to significantly improve electronic storage. FeRAM uses a material with a ferroelectric property to store data. The ferroelectric material can be polarized in one of two directions, representing a binary state. The polarization is retained even when the power is turned off, allowing the data to be stored permanently. FeRAM has fast read and writes speeds and low power consumption, but its density is lower than other types of memory, and its endurance is limited. RRAM uses a material with variable resistance to store data. When a voltage is applied to the material, its resistance changes, allowing data to be stored by changing the resistance. RRAM has fast read and writing speeds, high endurance, and low power consumption.

Among the emerging NVM technologies, RRAM is gaining traction because of its advantages over other technologies [6]. One of the main advantages of RRAM is its high speed. RRAM can switch on and off very quickly, which makes it ideal for use in applications that require fast read and write speeds. In comparison, FeRAM and MRAM have slower switching times. Another advantage of RRAM is its low power consumption. RRAM uses much less power than other non-volatile memory technologies, which makes it a more energy-efficient option. This is especially important for mobile devices and other battery-powered applications. RRAM also has a higher storage density than other non-volatile memory technologies. RRAM can be fabricated using smaller feature sizes than FeRAM and MRAM, allowing more data to be stored in a given area. PCRAM has a similar storage density to RRAM, but it has slower switching times and higher power consumption. According to the advantages, over the years, researchers have continued to refine and improve RRAM technology, developing new materials and manufacturing techniques that have made RRAM more practical and effective for modern applications.

One of the most promising forms of RRAM is based on boron nitride (BN) due to its advantages for aerospace applications, such as a wide-bandgap semiconductor with high thermal conductivity and excellent mechanical and chemical stability [7,8]. In detail, BN has a high thermal conductivity, which makes it an excellent candidate for use in heat dissipation applications, such as thermal management of electronic devices, and for use as a substrate for high-power electronic devices [9,10]. Secondly, BN is an excellent insulator, which makes it ideal for use in high-voltage applications, such as power distribution systems [11]. Thirdly, BN has a high strength-to-weight ratio and excellent mechanical stability [12], which makes it a suitable material for use in lightweight structures for aerospace and drone applications. BN has excellent thermal stability and is therefore well-suited for high-temperature applications. According to these advantages, RRAMs based on BN materials have emerged as promising candidates for embedded NVM applications in aerospace and drones [13,14,15]. The advantages of using BN-based RRAM for aerospace and drones are numerous. Firstly, BN-based RRAM offers low power consumption and high density [16,17], which is critical for the development of lightweight and energy-efficient aerospace and drone systems. Secondly, BN-based RRAM is highly reliable and robust, which is essential for operating these systems in challenging environments [18,19]. Thirdly, integrating BNs with RRAM allows for developing neural network models with reduced memory footprint, faster inference speed, and lower power consumption, making them ideal for deployment in aerospace and drone applications [20,21,22].

According to the above advantages, BN-based RRAM has been widely studied. For example, Jun Ge et al. reported low operating voltage below 500 mV and a large on/off ratio using hexagonal-BN (h-BN) [23]. However, one of the main challenges in BN-based RRAM is the presence of defects and trap states, which can degrade device performance and reliability for aerospace and drone applications.

Hydrogen annealing has been proposed as a promising technique to mitigate these issues. Hydrogen annealing involves exposing a material to hydrogen gas at high temperatures to react with dangling bonds or other defects, which can passivate bulk traps, and interface traps, and thereby improve the electrical properties of the material [24,25]. Hydrogen annealing is believed to improve the stability of RRAM devices by passivating defects in the metal oxide layer. This passivation occurs when hydrogen ions diffuse into the oxide layer and react with defects, such as oxygen vacancies. This process can reduce the number of defects in the oxide layer, making the formation and dissolution of conductive filaments more predictable and stable [26,27]. However, BN-based memory with hydrogen heat treatment has not been sufficiently studied.

In this paper, we propose BN-based RRAM with hydrogen annealing that has the potential to provide a unique combination of properties, including high endurance, low power consumption, and excellent retention characteristics, which make it an attractive option for aerospace and drone applications. Our proposed approach involves fabricating RRAM devices using h-BN as the active layer and hydrogen annealing the devices at a high temperature. We then characterized the electrical properties of the devices and compared them to those not hydrogen-annealed. Our results show that the hydrogen-annealed devices had better electrical performance than the non-annealed devices. Specifically, the hydrogen-annealed devices exhibited a larger on/off ratio and more stable characteristics than non-annealed devices. The improved performance can be attributed to the passivation of defects (or trap states) by the hydrogen annealing process.

## 2. Materials and Methods

The experimental procedure started with the deposition of 16 nm BN on a 100 nm Pt-coated substrate, which served as the bottom electrode (BE). The deposition was conducted using RF sputtering at room temperature, with a carrier gas concentration of 20 sccm argon (Ar). Before the gas was purged, the chamber’s base pressure was set to 20 mTorr. The deposition was then performed at a working pressure of 5 mTorr. Next, titanium (Ti) was deposited on the BN layer using RF sputtering and a mask. The deposition parameters used for Ti were kept the same as those for BN. To investigate the effect of hydrogen annealing, the device was annealed in an Ar/hydrogen environment using rapid thermal annealing (RTA, MILA-5000, ULVAC, Chigasaki, Japan) at 600 °C for 60 s. After the annealing process, the device was allowed to cool to room temperature.

As part of the experimental procedure, reference samples were also fabricated without any annealing and with nitrogen annealing. These samples were prepared in the same way as the other samples, with 16 nm BN deposited on a 100 nm Pt-coated substrate using RF sputtering at room temperature with a carrier gas concentration of 20 sccm Ar. The Ti layer was deposited on the BN layer using RF sputtering and a soft mask, with the same deposition parameters as for the other samples. In the case of the device with nitrogen annealing, the device was annealed in a nitrogen environment using RTA at 600 °C for 60 s.

To measure the current–voltage (*I–V*) characteristics of the sample, a Keithley 4200A-SCS parameter analyzer (Keithley Instruments, Solon, Ohio, USA) was used. The measurements were carried out in a vacuum chamber under ambient conditions. The *I–V* curves were obtained by applying a voltage sweep in steps of 0.01 V.

To determine the layer thickness and elemental composition of the BN, high-resolution transmission electron microscopy (HR-TEM) analysis was performed using a JEM-2100F (JEOL, Akishima, Tokyo, Japan). The sample was prepared by thinning the deposited film to electron transparency using a focused ion beam (FIB) milling technique using a Quanta 3D FEG (FEI, Hillsboro, Oregon, USA). The thickness of the sample was determined from the HR-TEM images, and the elemental composition was determined using energy-dispersive X-ray spectroscopy (EDS, Oxford Instrument, Oxfordshire, England).

## 3. Results and Discussions

**Figure 1:**
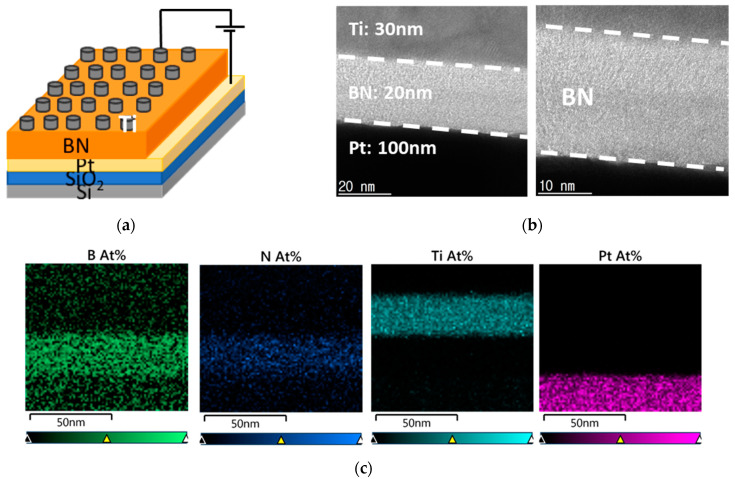
(**a**) schematic illustration, (**b**) HR-TEM, and (**c**) EDS results of proposed BN-based RRAMs.

Figure 1a is a schematic of the proposed device. It is a typical MIM structure with BN sandwiched between Ti and Pt. To check the crystallinity of the sample after heat treatment of BN at 600 °C for 60 s, HR-TEM was taken as shown in Figure 1b, and it was found that 20 nm of amorphous BN was deposited. Figure 1c shows the EDS result, indicating that the layer comprises B and N, where the ratio of B to N was 2:1.

**Figure 2:**
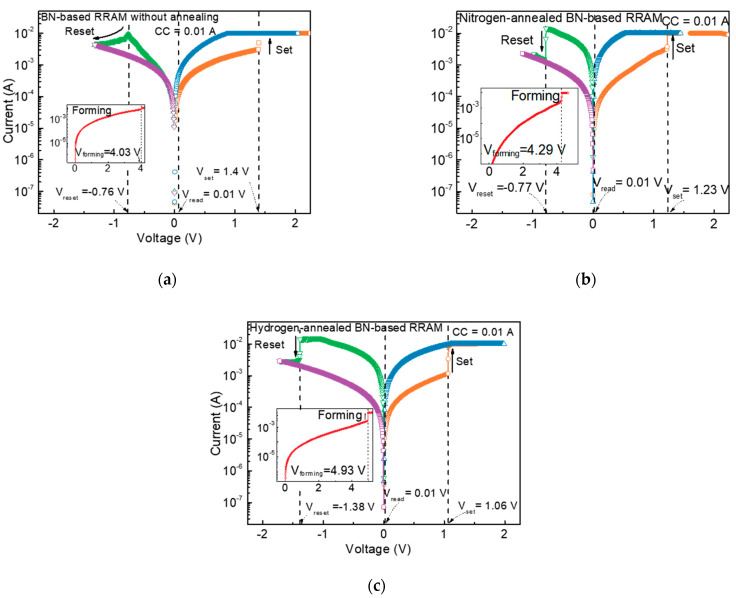
Resistive switching characteristics of (**a**) non-annealed, (**b**) nitrogen, and (**c**) hydrogen-annealed BN-based RRAM. In each figure, the inset shows the formation characteristic of the initial CFs.

To analyze the effect of hydrogen annealing on BN-based RRAMs, the resistance switching characteristics of BN-based RRAMs before annealing, after nitrogen annealing, and after hydrogen annealing were analyzed, respectively. Figure 2a–c shows the *I–V* curves of the BN-based RRAMs, indicating that all BN-based RRAMs clearly manifest a bipolar RS phenomenon in which the resistance of BN changes depending on the direction of current flow. Initially, in the case of BN-based RRAMs, BN has a high resistance and acts as an insulator. When a voltage is applied to BN, a current abruptly increases at a specific voltage due to the formation of a conductive path. This process is known as “forming” and is typically carried out at a voltage higher than the set voltage. The forming was taken in direct current (DC) mode and initiated the sweeping voltage in the positive direction. By setting the compliance current (CC) of 0.01 A, the first positive sweep resulted in the forming process at 4.03, 4.29, and 4.93 V for non-annealed, nitrogen-annealed, and hydrogen-annealed RRAM because of the primary formation of conduction filament as shown in the inset of Figure 2a–c. After the initial forming, to obtain the RS characteristic, the DC bias was swept from a negative to a positive direction, as shown in Figure 2a–c. When sweeping the DC bias in the negative direction, the change in the resistance state from low resistance state (LRS) to high resistance state (HRS) is observed at −0.76, −0.77, and −1.38 V for non-annealed, nitrogen-annealed, and hydrogen-annealed BN-based RRAM, which is called reset voltage. Subsequently, when we swept the DC voltage in the positive direction, the current was abruptly increased at 1.4, 1.23, and 1.06 V for non-annealed, nitrogen-annealed, and hydrogen-annealed devices, and the above process is called the set process. Then, as DC bias was applied again in the positive direction, the increased current was observed. These *I–V* curves are due to the conductivity mechanism being based on the creation and destruction of boron-vacancy-based conductive filaments [28]. During the formation operation, a high voltage is applied to the top electrode, and boron ions start to move toward TE, leading to the formation of boron vacancies. The boron vacancies can act as conductive paths between the TE and BE, facilitating electron transport through the BN, and resulting in the formation of a LRS in the devices. During the reset operation, a high negative voltage is applied to the bottom electrode, leading to the rupture of the conductive filaments, resulting in a HRS in the devices. During the set operation, a high positive voltage is applied to the top electrode, causing the boron vacancies to aggregate and form conductive filaments, resulting in the device transitioning to the LRS. The reason the forming voltage increases with annealing and hydrogen treatment seems to be crystallization and hydrogen passivation. The reason the set and reset voltages change after initial forming is that the density of the defect decreases after crystallization and hydrogen heat treatment. The correlation between the heat treatment, the heat treatment atmosphere, and the defect is discussed in detail in the section describing the conductivity mechanism.

The on/off ratio is an important parameter in RRAM devices as it determines their ability to reliably store and retrieve information. To evaluate the on/off ratio, we compared resistance differences between HRS and LRS at the read voltage (V_read_) of 0.1 V for non-annealed, nitrogen-annealed, and hydrogen-annealed devices. As a result, we obtained about 10^1^, 1.5 × 10^1^, and 10^2^ for the devices, respectively, indicating that the hydrogen-annealed BN-based RRAM shows enhanced on/off ratios due to the crystallization effect. The material’s resistance can be changed by applying a voltage to it, which causes the formation and dissolution of conductive filaments. However, the presence of defects and impurities in the material can lead to unwanted leakage currents, which can reduce the on/off ratio of the device. The hydrogen annealing process can help reduce the number of defects in BN-based RRAM, which can reduce leakage currents and increase the on/off ratio of the device, which is consistent with other papers [24,29].

**Figure 3:**
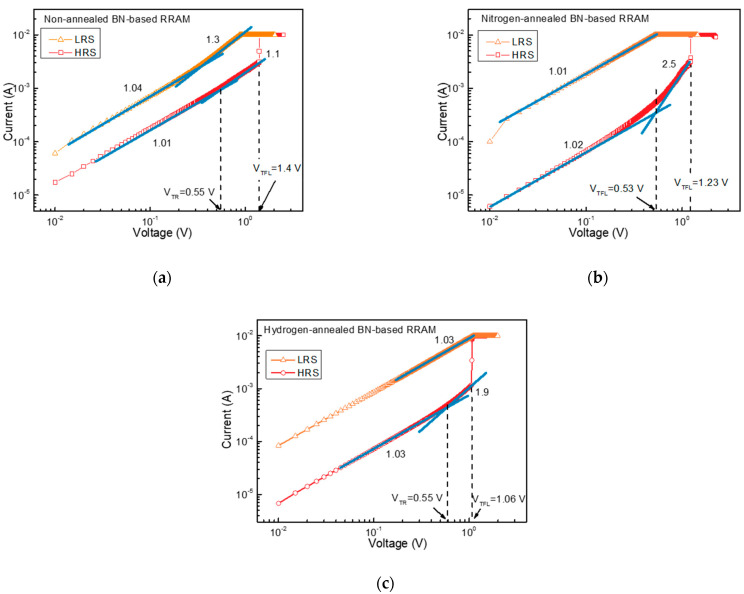
*I*–*V* characteristics on a double logarithmic scale in the positive voltage region of (**a**) non-annealed, (**b**) nitrogen-annealed, and (**c**) hydrogen-annealed BN-based RRAMs.

To further understand the effects of crystallization and hydrogen annealing on the BN-based RRAM, *I–V* curves for the devices are plotted and fitted with space charge limited conduction mechanism (SCLC), Fowler–Nordheim (FN) tunneling, Poole Frenkel, and thermionic emission conduction mechanism. Consequently, the *I–V* curves of both devices are well-matched with the SCLC mechanism. Figure 3a–c show *I–V* curves replotted in a double logarithmic scale under a cycle in the positive voltage region for devices. The yellow and red dots show the measured data, while the blue solid straight lines show the SCLC fitting results. The fitting results are consistent with the measured data, so the conduction mechanism of the proposed BN-based RRAMs is SCLC. In the low voltage region below 0.55 V, the current density follows Ohm’s law and is proportional to the applied voltage, leading to a linear *I–V* relationship. However, in the high voltage region above 0.55 V, the slope was increased to 1.1, 2.5, and 1.9 for non-annealed, nitrogen-annealed, and hydrogen-annealed BN-based RRAMs, leading to a non-linear *I–V* relationship. These behaviors of the current in different voltage regions, as shown above, fits well with the SCLC mechanism. The SCLC mechanism is modeled using the following formula:

(1) $$J=\frac{\theta }{\theta +1}\times \frac{9}{8}\varepsilon_{0}\varepsilon_{r}\mu \frac{V^{2}}{L^{3}}$$

where $\theta =(\frac{N_{c}}{N_{t}})exp(\frac{E_{c}}{E_{t}})/KT$ demonstrates the ratio of free to trapped electrons. Here, *N_c_* and *N_t_* are defined as the effective density of states present in the conduction band and the number of effective electron traps. *ε*_0_, *ε_r_*, and *μ* correspond to the permittivity of free space, static dielectric constant, and electron mobility, respectively, *V* is the applied voltage and *L* is the thickness of the BN film. In SCLC, transition voltage (V_TR_) and trap-filled limit voltage (*V_TFL_*) refer to two important parameters that can be derived from the *I–V* curve and provide information about the properties of BN. In the low voltage region below V_TR_, the electric field is uniformly distributed over BN, and there are no space charge regions in BN. Thus, current density follows Ohm’s law. When the electric field is increased to a higher injection, the trap sites in the BN that were not filled at low voltage are filled and the space charge region is generated. When the applied voltage exceeds V_TR_, the time it takes for externally injected charge carriers to travel through BN becomes very short. At this point, the number of thermally generated charge carriers in BN is no longer sufficient to relax or reduce the transit time of the externally injected carriers. This leads to a significant increase in the current density as the externally injected carriers move through the material more quickly and with less interference from the thermally generated carriers. As a result, the Fermi level in the BN increases and becomes higher than the trapping level, resulting in all the traps in the dielectric film being filled. The voltage at this point is V_TFL_ and can be calculated using the following formula:

(2) $$V_{TFL}=\frac{qN_{t}L^{2}}{2\varepsilon }$$

where *q* and *ε* are the electric charge and the static dielectric constant. To calculate *N_t_*, the static dielectric constant of the amorphous BN was assumed to be 1.88 [30]. As a result, for non-annealed, nitrogen-annealed, and hydrogen-annealed BN-based RRAM, *N_t_* was obtained 2.21, 2.03, and 1.67 × 10^13^/cm^3^, indicating that the annealing and the hydrogen passivation reduced bulk and interface traps, occurring the changes of the forming, set, and reset voltages.

**Figure 4:**
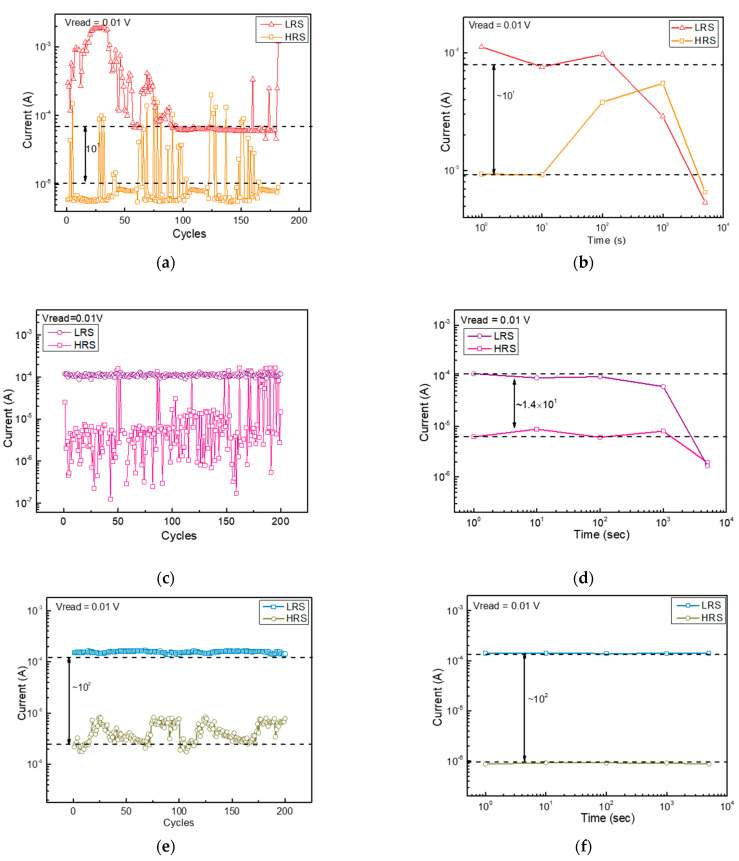
(**a**) Endurance and (**b**) retention characteristics of non-annealed BN-based RRAM. (**c**) Endurance and (**d**) retention characteristics of nitrogen-annealed RRAM. (**e**) Endurance and (**f**) retention characteristics of hydrogen-annealed RRAM.

To further demonstrate the reliability of the devices, endurance, and retention measurements were performed. In the case of non-annealed BN-based RRAM, 180 endurance cycles were measured, which appear very unstable from the start, and prominent current fluctuations are observed, as shown in Figure 4a. In the case of retention, the non-annealed BN-based RRAM showed very unstable results, and LRS and HRS started to overlap after 1000 s, as shown in Figure 4b. In the case of the nitrogen-annealed device, similar to the untreated sample, the endurance characteristics deteriorated with the increasing number of DC cycles, increasing the number of times it failed to set or reset (Figure 4c). In addition, the retention characteristics to maintain the LRS decreased, as shown in Figure 4d. On the other hand, hydrogen-annealed BN-based RRAM showed very stable endurance characteristics of 200 cycles and retention of 5000 s, as shown in Figure 4e,f.

**Figure 5:**
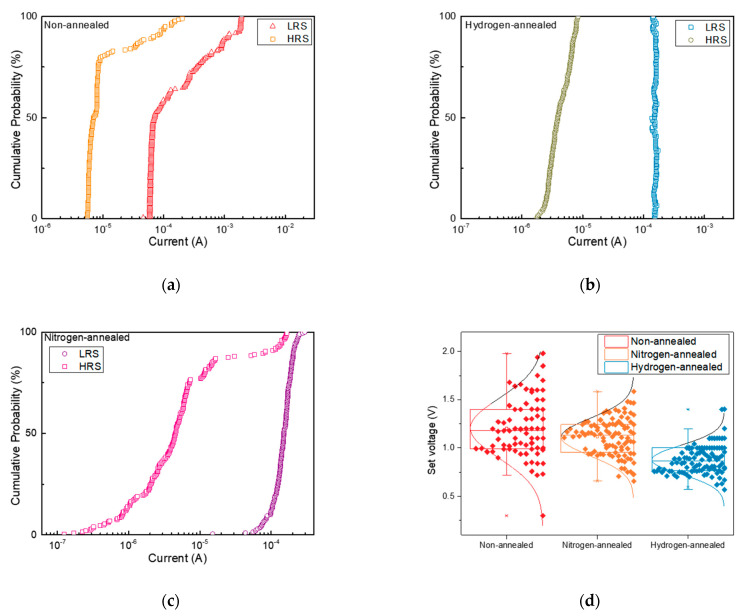

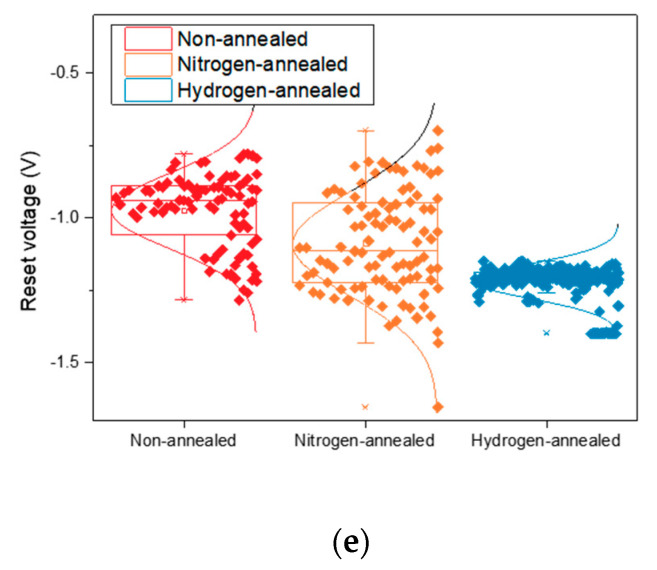
Cumulative characteristics of (**a**) non-annealed, (**b**) nitrogen-annealed, and (**c**) hydrogen-annealed BN-based RRAMs. (**d**) Set and (**e**) reset voltage distributions of the devices.

The commutative probability of LRS and HRS refers to the probability of the device switching between the two resistance states after multiple write/read cycles. If the device has a high probability of switching between LRS and HRS, it can result in data errors or data loss, which can negatively impact the device’s reliability. These results indicate that no obvious fluctuation is observed during the measurements which further confirms the improvements in the memory results. Figure 5a–c show the commutative probability by the resistance states of the devices. In the non-annealed and nitrogen-annealed cases, we observe that the HRS increases up to the initial LRS, which means that the probability of introducing errors or data loss is high. On the other hand, we observed that the ratio of LRS and HRS was well maintained in the case of a hydrogen-annealed device, indicating that the proposed hydrogen-annealed BN-based RRAM can secure stability for aerospace applications.

The distribution of set and reset voltages refers to the range of voltages required to switch the device between the LRS and HRS states. If the set and reset voltages have a wide distribution, a higher voltage should be applied to achieve a reliable set state, as the set may not occur when a low SET voltage is applied. Therefore, the wide distribution of the voltages can result in higher power consumption and lower device endurance due to the need for higher voltages to switch the device. This can also affect the device’s reliability over time. Therefore, we also evaluate the set-up and reset voltage distributions on 100 devices in non-annealed, nitrogen-annealed, and hydrogen-annealing, respectively, as shown in Figure 5d,e. In addition, we summarize the switching parameters of our fabricated devices with hydrogen-annealing and compare them to published data for a-BN and h-BN devices in Table 1. The standard deviation value of the set voltage is noteworthy. The non-annealed and nitrogen-annealed BN-based RRAMs exhibit standard deviations of 0.3 and 0.28, indicating the operational variability of the device. This can result in inconsistent device performance. However, the standard deviation value decreased to 0.14 for devices with hydrogen annealing, which is the lowest value compared to other studies. This decrease results from hydrogen-annealing that eliminates defects that can impact the performance of BN-based RRAM. As a result, we commonly observed the narrow distribution for hydrogen-annealed BN-based RRAM, indicating that the hydrogen-annealed devices can be not only low power consumption but also highly reliable for aerospace applications.

## 4. Conclusions

We obtained an on/off ratio of 100 on the hydrogen-annealed BN-based RRAM, which is 10 times higher than the non-annealed BN-based RRAM. Furthermore, we have demonstrated that hydrogen annealing has the effect of lowering not only the interface trap but also the bulk trap, thus changing the forming, reset, and set voltages. The device’s performance was maintained even after 200 DC cycling, and the stored resistance state did not change for 10,000 s. Furthermore, the distribution of the set and reset voltages was quite thin. The hydrogen annealing technique proposed in this paper not only secured the stability of BN-based RRAM but also secured its low-power operation characteristics, which will be a stepping-stone for aerospace memory devices.

## Figures and Tables

**Table 1:** Comparison of RRAM performance of a-BN based RRAM annealed in hydrogen with the published BN-based devices.

Device Structure	V_Set_ (V)	V_Reset_ (V)	Retention (s)	Endurance (#)	Standard Deviation of Set Voltage	Ref.
Ag/h-BN/Ag/p-Si	0.18	−0.39	10^3^	50	0.17	[31]
Au/h-BN/Ni	3	−1	~10^6^	~50	-	[32]
Ti/h-BN/Au	0.63	−0.44	3 × 10^3^	-	0.15	[33]
Ag/a-BN/Pt	0.4	−0.45	10^4^	10^4^	-	[34]
Ti/a-BN/Pt with hydrogen annealing	1.06	−1.38	>10^4^	200	0.14	In this work

## Data Availability

The data presented in this study are available on request from the corresponding author.
